# High-Sugar Diet Disrupts Hypothalamic but Not Cerebral Cortex Redox Homeostasis

**DOI:** 10.3390/nu12103181

**Published:** 2020-10-18

**Authors:** Ewa Żebrowska, Adrian Chabowski, Anna Zalewska, Mateusz Maciejczyk

**Affiliations:** 1 Department of Physiology, Medical University of Bialystok, 2c Mickiewicza Street, 15-233 Bialystok, Poland; adrian@umb.edu.pl; 2 Experimental Dentistry Laboratory, Medical University of Bialystok, Sklodowska 24a Street, 15-274 Bialystok, Poland; azalewska426@gmail.com; 3 Department of Hygiene, Epidemiology and Ergonomics, Medical University of Bialystok, 15-233 Bialystok, Poland

**Keywords:** high-sugar diet, oxidative stress, oxidative damage, cerebral cortex, hypothalamus

## Abstract

Despite several reports on the relationship between metabolic and neurodegenerative diseases, the effect of a high-sugar diet (HSD) on brain function is still unknown. Given the crucial role of oxidative stress in the pathogenesis of these disorders, this study was the first to compare the effect of an HSD on the activity of prooxidative enzymes, enzymatic and non-enzymatic antioxidants, and protein oxidative damage in the brain structures regulating energy metabolism (hypothalamus) and cognitive functions (cerebral cortex). Male Wistar rats were randomly divided into two groups (*n* = 10)—control diet (CD) and high-sugar diet (HSD)—for 8 weeks. We showed a decrease in glutathione peroxidase and superoxide dismutase activity and an increase in catalase activity in the hypothalamus of HSD rats compared to controls. The activity of xanthine oxidase and NADPH oxidase and the contents of oxidation (protein carbonyls), glycoxidation (dityrosine, kynurenine and N-formylkynurenine) and protein glycation products (advanced glycation end products and Amadori products) were significantly higher only in the hypothalamus of the study group. The HSD was also responsible for the disruption of antioxidant systems and oxidative damage to blood proteins, but we did not show any correlation between systemic redox homeostasis and the brain levels. In summary, HSD is responsible for disorders of enzymatic antioxidant defenses only at the central (plasma/serum) and hypothalamic levels but does not affect the cerebral cortex. The hypothalamus is much more sensitive to oxidative damage caused by an HSD than the cerebral cortex.

## 1. Introduction

The 21st century is defined as an epidemic of metabolic diseases that not only significantly reduce the quality of life of patients but are also the main cause of death worldwide. The decreased sensitivity of peripheral cells and tissues to insulin, which is called insulin resistance (IR), contributes to the metabolic disorders in the course of obesity, type 2 diabetes and metabolic syndrome [1]. It is undisputed that the most important factors in the development of IR are a lack of physical activity and improper diet, mainly considering an increased intake of fats and sugars. Although glucose is the primary source of energy for all body cells, in the course of hyperglycemia (primarily postprandial), we can observe an intensified production of reactive oxygen species (ROS), enhanced protein glycation and the induction of polyol and hexosamine pathways [2,3]. The binding of advanced glycation end products (AGEs) to a specific receptor (RAGE) not only increases ROS production but also induces the production of proinflammatory cytokines by activating the NF-kB (nuclear factor kappa-light-chain-enhancer of activated B cells) and c-Jun pathways [4,5]. It is believed that long-term sustained high glucose levels after meals play a key role in the development of diabetic complications [3,6]. Moreover, recent studies have indicated a link between an increased carbohydrate supply as well as insulin resistance and a greater risk of stroke or increased incidence of neurodegenerative diseases such as mild cognitive impairment (MCI), Alzheimer’s disease (AD) and Parkinson’s disease (PD) [7,8,9,10]. These changes are thought to be caused by disturbances in redox homeostasis and oxidative stress (OS) [10,11,12]. The latter is defined as an oxido-reductive imbalance leading to the oxidation of cellular biomolecules and thus to disturbed cell metabolism [13]. The excessive ROS activity causes structural and functional changes in enzymatic and regulatory proteins, damage to cell membranes as well as DNA and the induction of apoptosis [14]. Interestingly, the brain is particularly exposed to OS. This is determined by the intensive oxygen metabolism, high content of polyunsaturated fatty acids and relatively low activity of antioxidant enzymes [15,16]. Therefore, OS may be one of the mechanisms responsible for damage to brain cells under increased sugar supply.

In our previous studies, we demonstrated that a high-sugar diet (HSD) both interferes with systemic redox homeostasis (plasma/serum) and induces oxidative stress at the salivary gland level [17]. Interestingly, redox imbalance was observed only in the submandibular glands, which indicates the occurrence of organ-dependent metabolic disorders under the influence of an HSD. However, the effect of a dietary high sugar supply on metabolic changes in the brain, particularly including the redox balance of the hypothalamus and cerebral cortex—i.e., the brain structures regulating energy metabolism/the metabolism of lipids and carbohydrates as well as cognitive functions—is still unknown. Given the increasing incidence of metabolic and neurodegenerative diseases and the key role of OS in the pathogenesis of these disorders, the aim of this study was to assess the activity of prooxidative enzymes, enzymatic and non-enzymatic antioxidative barriers, redox status and oxidative damage to proteins in the selected brain structures of rats fed a high-sugar diet. Additionally, we evaluated the effect of an HSD on the redox homeostasis of the plasma/serum as well as selected apoptosis parameters in the brains of rats.

## 2. Materials and Methods

### 2.1. Animal Treatment

The study was approved by the Bioethics Committee at the Medical University of Bialystok, Poland (protocol number 89/2015, 2015/109). The experiments were performed on male Wistar rats weighing 67–72 g. The animals were housed under standard conditions (20 ± 2 °C, 12 h light/12 h dark cycle) with free access to food and drinking water. After a 7-day adaptation period, the rats (*n* = 20) were randomly divided into two groups. The control group was fed a control diet (CD; Research Diets, Inc., New Brunswick, NJ, USA; D12450K) consisting of 70 kcal% carbohydrates (sucrose, 0 kcal; cornstarch, 2200 kcal; maltodextrin, 10,600 kcal), 20 kcal% protein and 10 kcal% fat. The HSD group was fed a high-sucrose diet (HSD; Research Diets, Inc., New Brunswick, NJ, USA; D12450B) composed of 70 kcal% carbohydrates (sucrose, 1400 kcal; cornstarch, 1260 kcal; maltodextrin, 10,140 kcal), 20 kcal% protein and 10 kcal% fat (Farley et al. 2003). The calorie content of both diets was the same and amounted to 3.85 kcal/g. The body weights of the animals and food intake were monitored weekly. The body mass index (BMI) was calculated with the formula BMI = body weight (g)/length^2^ (cm^2^) [18]. Rat length was measured from the tip of the nose to the anus. BMIs between 0.45–0.68 g/cm^2^ were considered normal values, whereas BMIs greater than 0.68 g/cm^2^ indicated obesity [18]. After 8 weeks, all rats were anesthetized following overnight fasting with an injection of sodium pentobarbital (80 mg/kg of body weight, intraperitoneally). Next, whole blood was collected from the abdominal aorta into heparinized tubes (to obtain plasma) and glass tubes (to obtain serum) and centrifuged (3000× *g*, 4 °C, 10 min). At the same time, the cerebral cortex and hypothalamus were collected, immediately freeze-clamped in liquid nitrogen and then stored at −80 °C until being assayed.

Immediately prior to the determinations, the plasma and brain tissues were slowly thawed at 4 °C. The brain tissues were homogenized (Omni TH, Omni International, Kennesaw, GA, USA) in ice-cold phosphate-buffered saline (PBS) (1:15) and sonicated (20 s, three times, 1800 J/sample; ultrasonic cell disrupter, UP 400S, Hielscher, Teltow, Germany). Then, the homogenates were centrifuged for 20 min at 5000× *g* (MPW Med Instruments, Warsaw, Poland), and the supernatants were used for further assays [19]. All the above-mentioned steps were conducted at 4 °C. The brain tissues were treated with a protease inhibitor (1 tablet/10 mL of PBS; Complete Mini Roche, France) enriched with an antioxidant (100 µL of 0.5 M butylated hydroxytoluene (BHT) in acetonitrile per 10 mL of PBS) [20].

### 2.2. Plasma Insulin and Glucose Determination

The blood glucose concentration was measured using the Accu-Chek glucometer (Bayer, Germany). The plasma insulin concentration was measured with a commercially available ELISA kit according to the manufacturer’s instructions (Abbot, Johnson City, NY, USA). The insulin sensitivity was assessed using the homeostasis model assessment of insulin resistance (HOMA-IR) = fasting insulin (U/mL) × fasting glucose (mM)/22.5 [20].

### 2.3. Prooxidant Enzyme Activity Determination

The activity of NADPH oxidase (NOX) was measured using a luminescence assay with lucigenin as a luminophore [21]. One unit of NOX activity is required to release 1 nmol of superoxide anion per one minute. Xanthine oxidase (XO) activity was estimated colorimetrically at 290 nm by measuring the increase in the absorbance of the released uric acid (UA) [22]. One unit of XO activity was defined as the amount of enzyme required to release 1 μmol of UA per one minute.

All the assays were performed with duplicate samples of the homogenates of brain samples. The absorbance/fluorescence was measured using an Infinite M200 PRO Multimode Microplate Reader (Tecan Group Ltd., Männedorf, Switzerland). The presented results were standardized to mg of total protein. The total protein concentration was measured using the bicinchoninic acid (BCA) method [23] using the Thermo Scientific PIERCE BCA Protein Assay Kit (Rockford, IL, USA).

### 2.4. Enzymatic and Nonenzymatic Antioxidant Determination

Glutathione peroxidase (GPx) activity was analyzed spectrophotometrically based on the reduction of organic peroxides by GPx in the presence of NADPH [24]. Cu–Zn superoxide dismutase-1 (SOD-1) activity was assayed spectrophotometrically by measuring the cytosolic activity of SOD by inhibiting the oxidation of adrenaline at 480 nm [25]. Catalase (CAT) activity was determined in triplicate samples by measuring the decomposition rate for hydrogen peroxide (H_2_O_2_) at 240 nm [26]. The uric acid (UA) concentration was measured spectrophotometrically using a commercial kit from BioAssay Systems, Harward, CA, USA (QuantiChromTM Uric Acid DIUA-250 kit), as instructed by the manufacturer.

All the assays were performed in duplicate samples both of the brain samples and of the serum. The results were standardized to one mg of total protein.

### 2.5. Glutathione Metabolism Determination

The concentrations of oxidized (GSSG) and reduced (GSH) glutathione were determined colorimetrically at 412 nm based on the enzymatic reaction between NADPH, DTNB (5,5′-Dithiobis-(2-Nitrobenzoic Acid)) and GR (glutathione reductase) [27]. For the determination of GSSG, after thawing and neutralization (to pH 6–7 with 1 M chlorhydrol triethanolamine, TEA), the samples were incubated with 2-vinylpyridine (to inhibit glutathione oxidation). The GSH concentration was calculated using the difference between the levels of total glutathione and GSSG. Redox (oxidation/reduction) status was calculated using the formula [GSH]^2^/[GSSG] [28]. The thiol group concentration was colorimetrically measured using Ellman’s reagent (DTNB) in 0.1 M phosphate buffer, pH 8.0 [29]. The total thiol level was measured at 412 nm using GSH as a standard.

All the assays were performed with duplicate samples both of the brain samples and of the plasma. The results were standardized to one mg of total protein.

### 2.6. Redox Status Determination

Total antioxidant capacity (TAC) was estimated spectrophotometrically using 2,2-azinobis-3-ethylbenzothiazoline-6-sulfonic acid radical cation (ABTS*^+^) [30]. Changes in the absorbance of the ABTS*+ were determined at a 660 nm wavelength, and TAC was calculated from the calibration curve for Trolox (6-hydroxy-2,5,7,8-tetramethylchroman-2-carboxylic acid). Total oxidant status (TOS) was determined spectrophotometrically based on the oxidation of Fe^2+^ to Fe^3+^ in the presence of oxidants contained in the sample [31]. The absorbance was observed at 560/800 nm wavelengths. The oxidative stress index (OSI) was calculated using the formula OSI = TOS/TAC × 100 [32].

All the assays were performed with duplicate samples both of the brain samples and of the plasma. The results were standardized to one mg of total protein.

### 2.7. Oxidative Damage Determination

Advanced glycation end product (AGE) content was analyzed by measuring AGE-specific fluorescence at 350/440 nm [33]. The formation of Amadori products was determined colorimetrically using the Nitro Blue Tetrazolium (NBT) assay [34]. The absorbance was measured at 525 nm with the use of the extinction coefficient for monoformazan (12,640 M^−1^ cm^−1^). The concentration of advanced oxidation protein products (AOPPs) was estimated spectrophotometrically at 340 nm by the measurement of the iodine ion oxidative capacity [35]. For AGE and AOPP determination in plasma, all samples were diluted in PBS (pH 7.2) at 1:5 (*v*/*v*) [36].

The protein carbonyl (PC) concentration was analyzed colorimetrically with the use of 2,4-dinitrophenylhydrazine (2,4-DNPH)’s reaction with carbonyl groups in the oxidatively damaged proteins [37]. The absorbance of the resultant hydrazone was measured at 355 nm. To calculate PCs, an absorption coefficient for 2,4-DNPH (22,000 M^−1^ cm^−1^) was used.

The contents of dityrosine, kynurenine, N-formylkynurenine and tryptophan were measured based on the characteristic fluorescence at 330/415, 365/480, 325/434 and 95/340 nm, respectively [38]. The results were normalized to the fluorescence of 0.1 mg/mL quinine sulphate in 0.1 M H_2_SO_4_ [39].

All the assays were performed with duplicate samples both of the brain samples and of the plasma. The results were standardized to one mg of total protein.

### 2.8. Apoptosis Marker Determination

The nitric oxide (NO) level was determined spectrofluorimetrically by measuring NO’s stable decomposition products NO_3_^−^ and NO_2_^−^ from the Griess reaction [40]. Changes in the absorbance were measured at 543 nm. The assay was performed with duplicate samples both of the brain samples and of the plasma. The results were standardized to one mg of total protein.

Caspase-3 (CAS-3, EC 3.4.22.56) activity in the brain samples was measured colorimetrically using Ac-Asp-Glu-Val-Asp-p-nitroanilide as a substrate [41]. The amount of p-nitroaniline released by CAS-3 was measured at a 405 nm wavelength. The results were standardized to one mg of total protein.

### 2.9. Statistical Analysis

GraphPad Prism 8 for MacOS (GraphPad Software, La Jolla, CA, USA) was used for statistical analysis. The Shapiro–Wilk test was used to test for normality, and the Levene test was conducted to test the homogeneity of the variance. Specific analyses included one-way ANOVA and the post hoc Tukey test for honestly significant differences (HSDs). Student’s t-test was also used. The multiplicity-adjusted *p* value was also calculated. The associations between various parameters were tested by Pearson’s correlation. The threshold for statistical significance was *p* < 0.05 (two-sided).

Sample size calculation was conducted a priori based on a previous preliminary study. GPx and CAT activity, TOS, AGEs, dityrosine and PC concentrations were taken for the calculations. α = 0.05 and power = 0.8 were assumed for all cases. The sample sizes were estimated to be *n* = 5 to 10, with most studies indicating a sample size of 7 to 9 is needed [42,43,44,45]. Therefore, in this study, we chose to use a sample size of *n* = 10 per group.

## 3. Results

### 3.1. General Characteristics of the Animals

Energy intake and food consumption were similar in animals fed the high-sucrose diet (HSD) and control diet (CD) for 8 weeks (Table 1). However, the final body weight of the HSD rats was raised (+15%) compared to that of the controls. The BMI of the HSD-fed animals was 0.7, which confirmed obesity [18]. The HSD altered glucose homeostasis, as the plasma fasting glucose, insulin concentration and HOMA-IR were also higher (+23%, +15% and +600%, respectively) (Table 1).

### 3.2. Enzymatic and Non-Enzymatic Antioxidants, Glutathione Metabolism, Redox Status, Oxidative Damage and Apoptosis Markers in Plasma/Serum

Among the analyzed enzymes, only GPx activity was higher (+115%) in the plasma of HSD rats, whereas CAT activity remained unchanged and the SOD level was even lower (−29%) compared to that in the control group (Table 2). The uric acid concentration in the plasma of the animals fed the high-sucrose diet was similar to that in the controls. Glutathione metabolism was not altered by the HSD diet since GSH, GSSG, the [GSH]^2^/[GSSG] ratio and the total glutathione levels remained unchanged after 8 weeks of the diet experiment compared to those in the control rats (Table 2). Only the content of thiol groups in HSD plasma was decreased (−22%) in comparison with that in CD rodents. TAC and TOS levels were significantly elevated (+33% and +251%, respectively) in the plasma of HSD rats, whereas the OSI remained unchanged compared to the controls (Table 2). All of the oxidative damage markers assayed in the plasma/serum were considerably higher in the HSD group (except for tryptophan, which was lowered: −34% when compared to the control). The levels of AGEs, Amadori products, AOPPs, PCs, dityrosine, kynurenine and N-formylkynurenine were higher in the plasma of HSD rats compared to the controls (+37%, +65%, +138%, +414%, +34%, +28% and 42%, respectively). The NO content remained unchanged by the high-sugar feeding (Table 2).

### 3.3. Prooxidant Brain Enzymes

**Figure 1:**
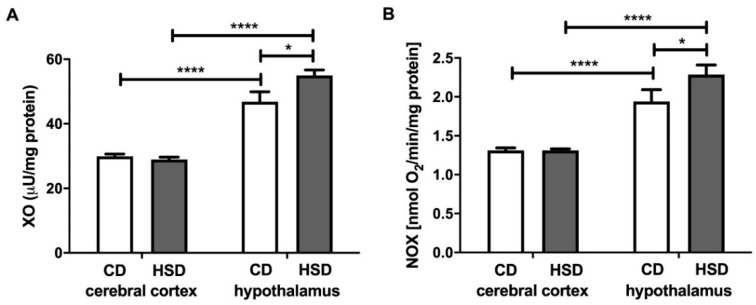
Prooxidant brain enzymes (xanthine oxidase (**A**) and NADPH oxidase (**B**)) in the control and HSD-fed rats. Values are means ± SEMs; *n* = 10, * difference statistically significant at *p* < 0.05 and **** *p* < 0.0001. CD—control diet; NOX—NADPH oxidase; HSD—high-sucrose diet; XO—xanthine oxidase.

The activity of xanthine oxidase in the hypothalamus was significantly higher (+17%) in HSD rats than in CD animals, whereas in the cerebral cortex, it remained unchanged (Figure 1). Furthermore, we observed a considerable difference in the activity of XO between both studied brain structures in CD as well as HSD animals (+56% and +90%; hypothalamus vs. cerebral cortex). NADPH oxidase activity was higher only in the hypothalamus of HSD animals (+18%) when compared to controls; however, its activity was markedly higher in the hypothalamus compared to the cerebral cortex both in the control and HSD animals (+48% and +74%, respectively, in the hypothalamus vs. cerebral cortex) (Figure 1).

### 3.4. Enzymatic and Non-Enzymatic Brain Antioxidants

**Figure 2:**
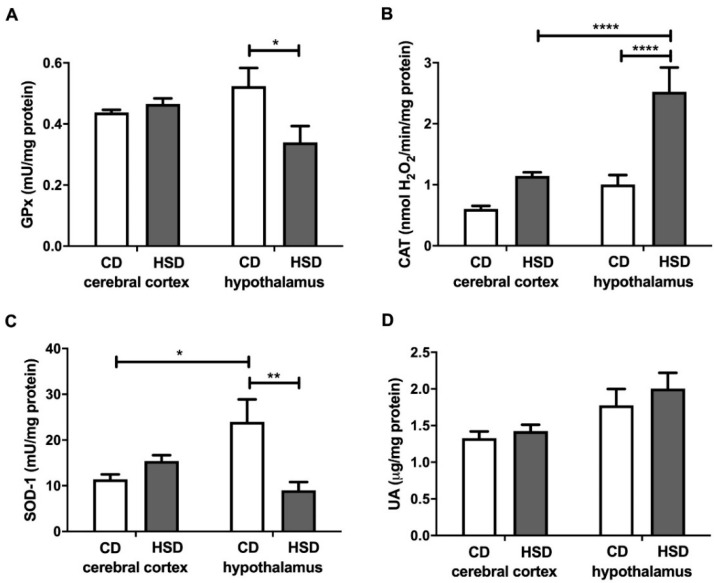
Enzymatic and non-enzymatic brain antioxidants (glutathione peroxidase (**A**), catalase (**B**), Cu-Zn-superoxide dismutase-1 (**C**) and uric acid (**D**)) in the control and HSD-fed rats. Values are means ± SEMs; *n* = 10 * difference statistically significant at *p* < 0.05, ** *p* < 0.005 and **** *p* < 0.0001. CAT—catalase; CD—control diet; GPx—glutathione peroxidase; HSD—high-sucrose diet; SOD-1—Cu-Zn-superoxide dismutase-1; UA—uric acid.

GPx and SOD-1 activities in the hypothalamus were lower in HSD-fed animals than in the controls (−35% and −62%, respectively), whereas in the cerebral cortex, their values remained unchanged by the diet (Figure 2). SOD-1 activity was higher in the hypothalamus (+110%) than in the cerebral cortex, but only in CD rats. CAT activity was significantly higher (+150%) only in the hypothalamus of the HSD group, and higher (+121%) in the hypothalamus of HSD rats compared to the cerebral cortex. The uric acid concentrations both in the cerebral cortex and hypothalamus of HSD rats did not differ from those in the control rats (Figure 2).

### 3.5. Brain Glutathione Metabolism

**Figure 3:**
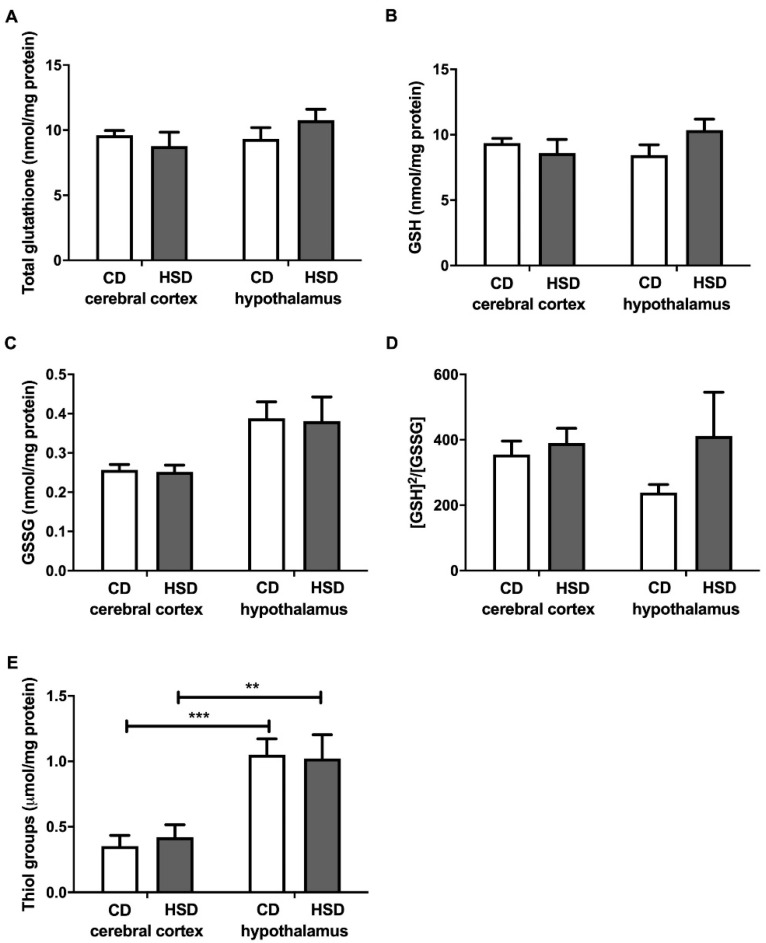
Brain glutathione metabolism (total glutathione (**A**), reduced glutathione (**B**), oxidized glutathione (**C**), the oxidation/reduction ratio (**D**) and thiol groups (**E**)) in the control and HSD-fed rats. Values are means ± SEMs; *n* = 10 * differences statistically important at *p* < 0.05, ** *p* < 0.005 and *** *p* < 0.0005. CD—control diet; GSH—reduced glutathione; [GSH]^2^/[GSSG]—the oxidation/reduction ratio; GSSG—oxidized glutathione; HSD—high-sucrose diet.

Total glutathione, GSH, GSSG and the [GSH]^2^/[GSSG] ratio did not differ in both the cerebral cortex and hypothalamus in any of the studied groups (Figure 3), whereas the content of thiol groups was generally higher in the hypothalamus compared to the cerebral cortex (+199% and +143% for CD and HSD, respectively).

### 3.6. Brain Redox Status

**Figure 4:**
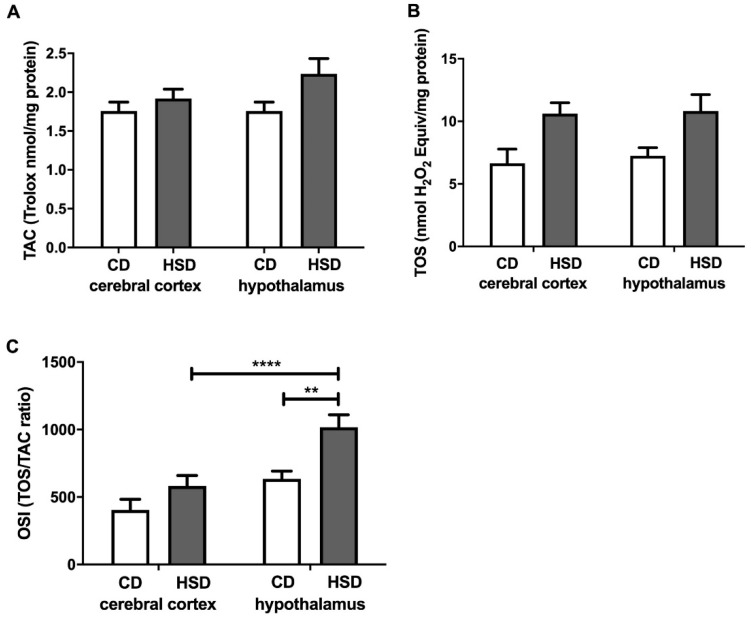
Brain redox status (total antioxidant capacity (**A**), total oxidative status (**B**) and oxidative stress index (**C**)) in the control and HSD-fed rats. Values are means ± SEMs; *n* = 10, ** differences statistically important at *p* < 0.005 and **** *p* < 0.0001. CD—control diet; HSD—high-sucrose diet; OSI—oxidative stress index; TAC—total antioxidant capacity; TOS—total oxidative status.

TAC and TOS were similar in all of the examined groups (Figure 4). The OSI was considerably higher only in the hypothalamus (+60%) of HSD animals compared to the controls. In the hypothalamus of HSD animals, the OSI was higher (+75%) when compared to that in the cerebral cortex (Figure 4).

### 3.7. Brain Oxidative Damage Markers

**Figure 5:**
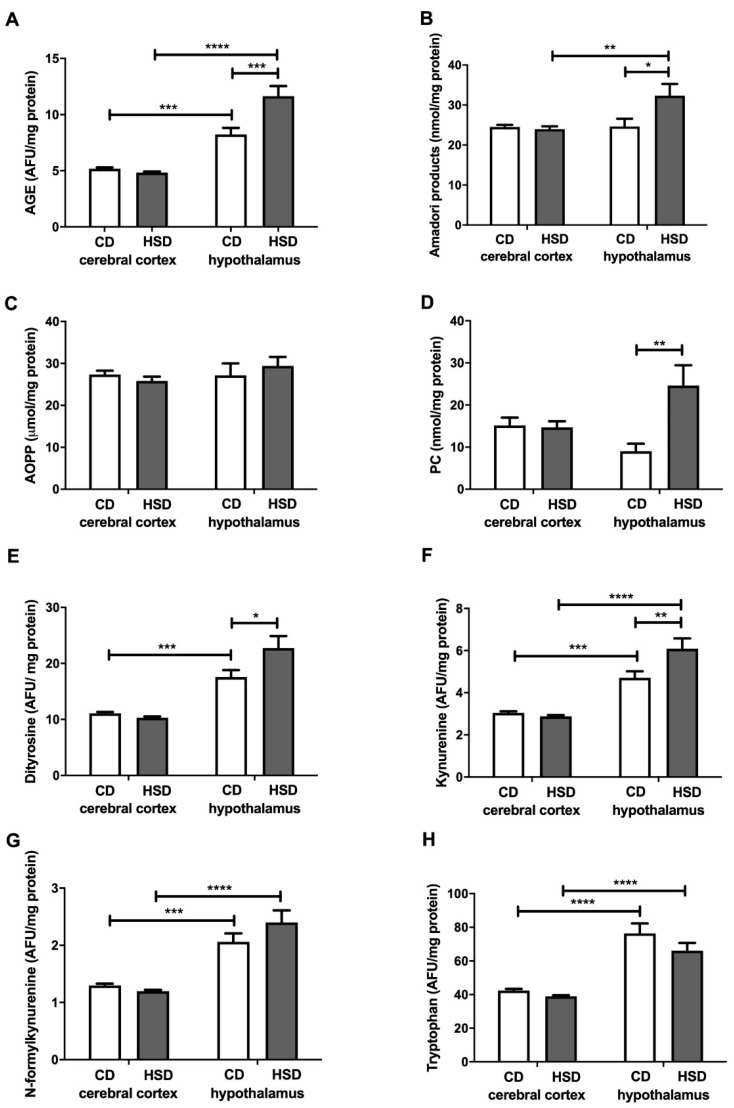
Brain oxidative damage (advanced glycation end products (**A**), Amadori products (**B**), advanced oxidation protein products (**C**), protein carbonyls (**D**), dityrosine (**E**), kynurenine (**F**), N-formylkynurenine (**G**) and tryptophan (**H**)) in the control and HSD-fed rats. Values are means ± SEMs; *n* = 10 * differences statistically important at *p* < 0.05, ** *p* < 0.005, *** *p* < 0.0005 and **** *p* < 0.0001. AGE—advanced glycation end products; AOPP—advanced oxidation protein products; CD—control diet; HSD—high-sucrose diet; PC—protein carbonyls.

The contents of all the assayed cerebral cortex damage markers in HSD rats were similar to those in the CD group, whereas in the hypothalamus, most of these markers were higher compared to in the controls: AGEs (+41%), Amadori products (+31%), PCs (+173%), dityrosine (+29%) and kynurenine (+29%) (Figure 5). The concentrations of N-formylkynurenine and tryptophan were higher in the hypothalamus than in the cerebral cortex (+59% and +80% in the CD group; +100% and 70% in HSD rats, respectively). The PC content was lower in the hypothalamus of HSD rodents vs. the controls (−63%) (Figure 5).

### 3.8. Brain Apoptosis Markers

**Figure 6:**
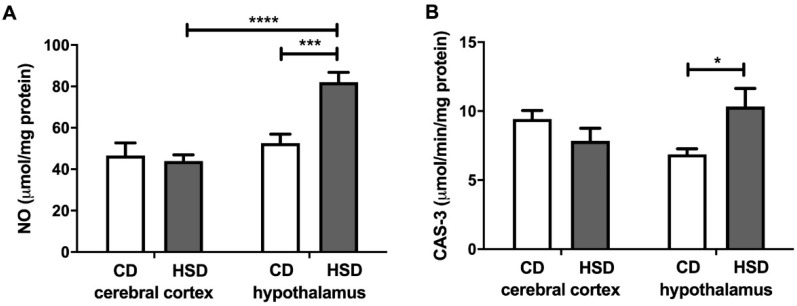
Brain apoptosis markers (nitric oxide (**A**) and caspase 3 (**B**)) in the control and HSD-fed rats. Values are means ± SEMs; *n* = 10 * differences statistically important at *p* < 0.05, *** *p* < 0.0005 and **** *p* < 0.0001. CAS-3—caspase 3; CD—control diet; HSD—high-sucrose diet; NO—nitric oxide.

NO and CAS-3 were higher only in the hypothalamus (+56% and +50%, respectively) of HSD rats when compared to the controls (Figure 6). Moreover, the NO content was significantly higher (+87%) in the hypothalamus of HSD animals compared to the cerebral cortex.

### 3.9. Correlations

In the hypothalamus of HSD rats, a high positive correlation between the activity of prooxidative enzymes (NOX and XO) and CAT activity was shown (*r* = 0.698, *p* = 0.05; *r* = 0.754, *p* = 0.029). NOX and XO were also positively correlated with AGE content (*r* = 0.856, *p* = 0.007; *r* = 0.87, *p* = 0.005). We did not demonstrate such relationships in the cerebral cortex of HSD animals or in either of the studied brain structures of the control animals.

We demonstrated a positive correlation between CAT activity and AGE (*r* = 0.65, *p* = 0.05) as well as PC (*r* = 0.955, *p* < 0.0001) content only in the hypothalamus of HSD rats.

We did not observe any correlation between any of the redox biomarkers assayed in the brain and serum/plasma.

## 4. Discussion

The latest studies indicate deleterious effects of an HSD on neurotransmission, neurogenesis and synaptogenesis [46,47,48]. However, there are no studies concerning brain redox homeostasis under high sugar intake. This study is the first to compare the effect of a high-sugar diet on redox homeostasis in the blood as well as hypothalamus and cerebral cortex of Wistar rats. We demonstrated that an 8-week HSD diet not only induces IR and systemic oxidative stress, but also disrupts redox balance at the brain level. Interestingly, the hypothalamus is much more sensitive to oxidative damage than the cerebral cortex.

It is difficult to conclude regarding the redox homeostasis solely on the basis of a single biomarker [49]. Therefore, in our study, we examined prooxidant enzymes, enzymatic and non-enzymatic antioxidants, and redox status as well as protein glycation and oxidation.

We showed that an 8-week HSD diet induces obesity, hyperglycemia, hyperinsulinemia, insulin resistance and systemic oxidative stress in rats. In the plasma/serum of rats from the study group, we observed disturbances of the enzymatic antioxidant barrier (↑GPx and ↓SOD-1) as well as higher oxidative damage to proteins (↑Amadori products, ↑AGEs, ↑PCs, ↑AOPPs, ↑glycoxidation products and ↓total thiols) compared to in rats fed a standard diet (Table 2). These observations are consistent with the results of other studies. Interestingly, we did not demonstrate any correlations between systemic redox homeostasis and changes at the level of the hypothalamus and cerebral cortex, which may indicate a different nature of redox imbalance.

Aerobic organisms have developed both enzymatic and non-enzymatic antioxidant systems in order to protect against ROS overproduction. However, the activity of brain antioxidant enzymes (compared to those in other tissues) is relatively low [15]. Our study showed a decreased activity of SOD-1 and GPx as well as higher CAT activity only in the hypothalamus of HSD rats (vs. the controls) (Figure 2). Although we did not directly evaluate the rate of ROS production (or H_2_O_2_ concentration), increased CAT activity may suggest an enhanced production of free radicals in the hypothalamus of the rats from the study group. Indeed, both CAT and GPx are involved in the cytotoxic decomposition of hydrogen peroxide, but CAT is active at high and GPx at low intracellular H_2_O_2_ concentrations [50,51].

Interestingly, we did not observe any changes in the total antioxidant capacity and total oxidant status or differences in glutathione metabolism between the studied brain structures. However, in the hypothalamus of HSD rats, we noted a significantly higher oxidative stress index (compared to in the controls), which indicates a shift in brain redox homeostasis towards oxidation reactions. Indeed, this parameter (OSI = TOS/TAC) predisposes the biological system to oxidative stress [52]. Moreover, the activity of cerebral prooxidative enzymes (↑NOX and ↑XO) was significantly higher in the hypothalamus of HSD rats compared to in the control group (Figure 1). A strong positive correlation between NOX and XO activity and CAT activity may suggest an adaptive response of the hypothalamus to increased ROS production triggered by HSD. It is well known that the strengthening of the enzymatic antioxidant barrier is the first line of defense against OS [53], and the decreased activity of SOD-1 and GPx in the hypothalamus of HSD rats is most likely caused by the exhaustion of antioxidant reserves under free radical overproduction (↑OSI).

The consequence of the disruption of antioxidant systems as well as increased activity of prooxidant enzymes is the increased oxidation of hypothalamic proteins (↑Amadori products, ↑AGEs, ↑PCs and ↑glycoxidation products). This hypothesis is confirmed by a positive correlation between the activity of antioxidant enzymes (CAT) and the products of oxidation (PCs) and glycation of proteins (Amadori products and AGEs) in the hypothalamus of HSD rats, and a positive correlation between the activity of prooxidant enzymes and AGEs. However, such changes were not observed in the cerebral cortex of rats from the study group.

The main cell components that undergo oxidation and glycation processes are proteins [54,55]. Interestingly, in a typical eukaryotic cell, over 70% of hydroxyl radicals react with proteins, and in the brain, this frequency may be even higher [55]. The brain is characterized by a high content of prooxidant metal ions, which are known to loosely bind to protein molecules [15,44]. Our study showed that an HSD increases both the oxidation (↑PC) and glycation (↑dityrosine, ↑kynurenine, ↑Amadori products and ↑AGEs) of hypothalamic proteins (Figure 5). Although the glycation of proteins is very slow under physiological conditions, glucose binding to proteins is much faster at high glucose concentrations [56,57]. However, it is not only the increased availability of carbohydrate and lipid substrates that may intensify this process; enhanced oxygen metabolism may also. This leads to glucose auto-oxidation as well as the increased formation of dicarbonyl compounds, which significantly raises the glycoxidation rate for proteins [2,58]. It has been demonstrated that protein oxidation and glycation lead to damage to amino acid residues, the breaking of polypeptide chains and the formation of cross-links, thus entailing the loss of the biological activity of modified proteins and their tendency to aggregate and accumulate [54,55,59]. This process is particularly important in neurodegenerative diseases (AD and PD) that are accompanied by an additional decrease in the activity of proteasomes responsible for damaged protein removal [60,61,62]. Interestingly, protein advanced glycation end products (mainly AGEs) may increase ROS production by inducing NOX activity as well as activating proinflammatory signaling pathways (mainly NF-kB) [5,63]. In our study, this was suggested by a positive correlation between NOX activity and AGE concentration in the hypothalamus of HSD rats. Thus, these conditions may lead to the boosted production of cytokines, chemokines and growth factors as well as increased expression of adhesion molecules (such as ICAM or VCAM). Furthermore, the accumulation of protein glycation products in the brain tissue may lead to cell death through apoptosis or necrosis [4,64]. It is noteworthy that both NO concentrations and caspase-3 activity were significantly higher in the hypothalamus of HSD rats compared to the controls (Figure 6). It is well known that, depending on the concentration, nitric oxide has different effects on the apoptosis process: an excess of NO decreases the ATP concentration as well as the potential of the internal mitochondrial membrane, which results in the inflow of calcium ions and apoptosis [65,66].

We did not observe increased carbonyl stress or apoptosis in the cerebral cortex of HSD rats. Moreover, the efficiency of the antioxidant barrier and the activity of prooxidant enzymes in the cerebral cortex did not differ significantly between the study and control groups. Therefore, we conclude that the hypothalamus is much more sensitive to HSD-induced oxidative damage. This organ, due to the synthesis and secretion of numerous hormones, regulates lipid and carbohydrate metabolism. Indeed, the hypothalamus is characterized by a high density of insulin receptors, which are involved in the central control of the metabolism of peripheral tissues as well as regulation of appetite and satiety [67,68]. However, changes in insulin transmission also lie at the root of many neurodegenerative diseases [11,60]. It is believed that cerebral oxidative stress (particularly mitochondrial dysfunction) may disrupt insulin signaling within the brain [69,70]. Thus, the results of our study may explain the fact that people who consume excessive amounts of carbohydrates in their diet are much more likely to develop obesity and cardiovascular complications than cognitive disorders [71,72]. Unfortunately, in our experiment, we did not perform cognitive tests that could have confirmed (or excluded) the occurrence of cognitive impairment in HSD rats. However, recent studies have indicated that the increased sensitivity of the hypothalamus to ROS may be caused by the different energy metabolism of brain structures, which additionally changes with age [73]. Nevertheless, this hypothesis requires further research and observation.

Our work, despite its undoubted advantages, also had certain limitations. We evaluated only the most commonly used redox and apoptosis biomarkers, so we cannot fully compare the effect of the HSD on redox homeostasis in the brain as well as plasma/serum. Furthermore, our study did not include the assessment of cognitive functions in rats, so we were unable to determine whether OS in the hypothalamus results from hyperglycemia or other metabolic disorders caused by the HSD. Therefore, this work is a starting point for further research, both experimental and clinical.

## 5. Conclusions

(i) A high-sugar diet disrupts redox homeostasis, not only at the systemic but also local (cerebral) level.

(ii) Disorders of the enzymatic antioxidant defense caused by an HSD were observed only at the central (plasma/serum) and hypothalamic levels. However, it is necessary to assess cognitive function in order to confirm or exclude the neuropathological disturbances caused by a high sugar intake.

(iii) The hypothalamus is much more sensitive to oxidative damage caused by an HSD than the cerebral cortex.

## Figures and Tables

**Table 1:** General characteristics of the control and high-sugar diet (HSD)-fed rats.

Parameter	CD	HSD
Body weight (g)	356 ± 11.47	411 ± 7.54 ***
BMI (g/cm^2^)	0.64 ± 0.024	0.70 ± 0.016 *
Food intake (g/day)	21.4 ± 1.32	21.8 ± 1.42
Energy intake (g/rat/week)	82.4 ± 5.07	84 ± 5.46
Glucose concentration (mg/dL)	137 ± 5.54	169 ± 5.01 ***
Insulin concentration (mU/mL)	0.18 ± 0.007	0.27 ± 0.03 *
HOMA-IR	2.73 ± 0.13	19.2 ± 0.95 ****

Values are means ± SEMs; *n* = 10 * difference statistically significant at *p* < 0.05, *** *p* < 0.0005, **** *p* < 0.0001. BMI—body mass index; CD—control diet; HOMA-IR—homeostatic model assessment of insulin resistance; HSD—high-sucrose diet.

**Table 2:** Effect of 8-week HSD on enzymatic and non-enzymatic antioxidants, glutathione metabolism, redox status, oxidative damage and apoptosis markers in plasma/serum of rats.

Parameter	CD	HSD
**Enzymatic and non-enzymatic antioxidants**		
GPx (mU/mg protein)	0.88 ± 0.09	1.9 ± 0.16 ***
CAT (nmol H_2_O_2_ min^−1^ mg protein^−1^)	4.52 ± 0.64	3.84 ± 0.69
SOD-1 (mU/mg protein)	43.5 ± 1.21	30.9 ± 1.1 ****
UA (µg/mg protein)	0.44 ± 0.05	0.41 ± 0.05
**Glutathione metabolism**		
Total glutathione (nmol/mg protein)	20.9 ± 1.28	16.5 ± 2.72
GSH (nmol/mg protein)	20.4 ± 1.27	16.0 ± 2.71
GSSG (nmol/mg protein)	0.50 ± 0.03	0.51 ± 0.03
[GSH]^2^/[GSSG]	882 ± 116	634 ± 172
Thiol groups (nmol/mg protein)	3.22 ± 0.19	2.51 ± 0.20 *
**Redox status**		
TAC (Trolox nmol/mg protein)	0.09 ± 0.002	0.12 ± 0.01 *
TOS (nmol H_2_O_2_ Equiv./mg protein)	0.79 ± 0.07	2.77 ± 0.31 ***
OSI (TOS/TAC ratio)	9.24 ± 0.93	21.65 ± 5.25
**Oxidative damage**		
AGEs (AFU/mg protein)	297 ± 6.17	408 ± 22.7 **
Amadori Products (nmol/mg protein)	11.1 ± 0.77	18.3 ± 1.01 **
AOPPs (μmol/mg protein)	6.2 ± 1.2	14.8 ± 1.3 **
PCs (nmol/mg protein)	0.70 ± 0.74	3.6 ± 0.56 **
Dityrosine (AFU/mg protein)	507 ± 37.03	681 ± 18.44 **
Kynurenine (AFU/mg protein)	330 ± 22.9	424 ± 22.0 *
N-formylkynurenine (AFU/mg protein)	270 ± 15.34	383 ± 15.75 **
Tryptophan (AFU/mg protein)	28.9 ± 1.12	19.04 ± 2.55 *
**Apoptosis markers**		
NO (μmol/mg protein)	21.5 ± 1.42	23.2 ± 2.83

Values are means ± SEMs; *n* = 10 * difference statistically significant at *p* < 0.05, ** *p* < 0.005, *** *p* < 0.0005 and **** *p* < 0.0001. AGEs—advanced glycation end products; AOPPs—advanced oxidation protein products; CAT—catalase; CD—control diet; GPx—glutathione peroxidase; GSH—reduced glutathione; [GSH]^2^/[GSSG]—the oxidation/reduction ratio; GSSG—oxidized glutathione; HSD—high-sucrose diet; NO—nitric oxide; NOX—NADPH oxidase; OSI—oxidative stress index; PCs—protein carbonyls; SOD-1—Cu-Zn-superoxide dismutase-1; TAC—total antioxidant capacity; TOS–total oxidative status; UA—uric acid; XO—xanthine oxidase.
